# Qualitative research of informal caregivers’ personal experiences caring for older adults with dementia in Lithuania

**DOI:** 10.1186/s13033-020-00428-w

**Published:** 2021-01-20

**Authors:** Ausrine Kontrimiene, Jolanta Sauseriene, Aurelija Blazeviciene, Gediminas Raila, Lina Jaruseviciene

**Affiliations:** 1 Department of Family Medicine, Lithuanian University of Health Sciences (LUHS), Mickeviciaus 9, 44307 Kaunas, Lithuania; 2 Department of Nursing and Care, Lithuanian University of Health Sciences (LUHS), Eivenių str. 4, 50161 Kaunas, Lithuania

**Keywords:** Informal care, Patient care, Mental health services, Primary health care, Social services, Older adults, Dementia

## Abstract

**Background:**

In many communities around the world, informal caregivers of older adults with dementia represent an essential, yet often underappreciated, source of long-term care. The present study aimed to determine the personal experiences of such caregivers, which could be instrumental for developing means of improving the quality of care for both care receivers and their informal caregivers.

**Methods:**

Five semi-structured focus-group discussions were held. The participants (n = 31) were all informal caregivers of older adults with dementia. The focus-group discussions were audio-recorded and transcribed verbatim. An inductive approach was used, and thematic data analysis was applied.

**Results:**

Four thematic categories were identified: learning caregiving through personal experience; implications of caregiving on social wellbeing; caregivers’ contradictory emotions regarding care delivery; and addressing challenges regarding care provision.

**Conclusions:**

This study revealed, among the informal caregivers, a variety of experiences, contradicting feelings, and problem-solving strategies relating to the care of older adults with mental disorders. Becoming an effective caregiver involves professional and psychological development. Developing caregiving skills, supportive environment and positive attitude can help facilitate providing care. Caregiving largely impacts the emotional, physical, and social wellbeing of the person; thus, comprehensive approaches are needed to prevent burnout and associated social disadvantages.

## Background

In low- and middle-income countries, a large amount of the care provided for individuals with chronic diseases and/or disabilities is administered by informal caregivers [1]. This trend is becoming particularly widespread in welfare states (e.g. the Netherlands), as a result of changes in citizens’ roles in society and the application of austerity measures [2, 3]. Informal care is indisputably valuable to the entire health-care system, as it incurs lower health- and social-care costs and is associated with lower rates of institutionalization for care-receivers [4]. Further, societal ageing increases informal caregivers’ value within the health system, especially those who are caring for older adults with dementia and Alzheimer’s disease: the hours of informal care provided to these patients worldwide is the equivalent of 40 million full-time jobs [5]. However, despite this huge contribution to the care of older adults with dementia, the burden of such informal caregiving is often under estimated, and means of maintaining this indispensable care resource remain understudied [6–8].

A recent study conducted in the Netherlands revealed that informal caregivers of people with dementia experience a higher caregiving burden than do regular-care caregivers [9]. Other studies have supported this, showing that informal caregiving for older adults with dementia has a negative effect on the caregivers themselves, such as by increasing loneliness, stress, and social isolation [10, 11]; negatively impacting mental and physical health outcomes [12–14]; and impeding financial potential [15]. Further, caregivers have been found to self-report lower levels of quality of life and health in general when compared to non-caregivers [1, 9] and to have chronic physical conditions [16].

In Lithuania, as in many Central and Eastern European countries, informal caregiving based on the Semashko model (which was originally developed during the Soviet Union years and distributed across constituent states) has traditionally played a significant role in long-term care provision [17]. The Semashko model represents a centralized health-care model in which patient care is mainly provided by district physicians and specialists [17]. However, Lithuania’s social-care services remain in the development stage and, at present, institutional care is the only form of social- and nursing-care service available for older adults [18, 19]. Lithuania, similar to several other countries in Eastern Europe, also has underdeveloped support schemes for caregivers, and only provides benefits for dependent persons; this situation has previously been described as inadequate [20]. Furthermore, studies of other countries have shown that the fragmentation of health and social care can cause inconsistencies in care provision and to negatively impact the use of existing related resources [6, 18, 20].

Research on health- and social-care environments in Lithuania commenced following the initiation of healthcare reforming the 1990s, after the collapse of the Soviet Union. However, these early studies were mostly confined to assessment of the basic needs of people with disabilities, with the role of informal caregivers being underappreciated or disregarded entirely. The experience of informal caregivers in Lithuania has been academically examined as recently as approximately 2006; however, informal caregivers of patients with dementia have only been considered in a very small number of studies, which were also small in scale [21, 22]. Nevertheless, these studies revealed that informal caregivers in Lithuania have a lack of clear information regarding their role and responsibilities, difficulties adapting to the home environment, a need to combine their work and caregiving, and a lack of support from other family members and institutions [21, 22].

Informal caregiving for older adults with dementia can be considered a sensitive and complex phenomenon that requires in-depth, large-scale analysis that encompasses a range of cultures and contexts. Further, development of national policies for long-term care for older adults with dementia requires an assessment of informal caregiving, as this service represents an essential aspect of the care system. The present study aimed to further clarify such caregivers’ experiences caregiving for older adults with dementia, which could be instrumental for identifying means of improving the quality of care provided for such adults and for highlighting the need for protective policies for their informal caregivers.

## Methods

### Study design

This study forms part of an ongoing large-scale, multi-directional, 3-year (2017–2020) project titled “Integrated Health Care for Senior Mental Health: Developing an Intersectoral Cooperative Care Model, “which is financed by the Lithuanian Research Council (S-MIP-17-121). Our research team aims to comprehensively identify the health-care- and social-care-related needs of Lithuania-based older adults with dementia and their family caregivers. Furthermore, we also seek to identify means of optimizing the use of available health- and social-care resources in a manner that improves such patients’ wellbeing. The underlying aim of the project is to improve health- and social-care provision for older adults with dementia and their informal caregivers by establishing horizontal cooperation links among professionals from different sectors. In particular, by focusing on both care receivers and informal caregivers, the issues identified in this area can be considered to be comprehensively representative.

The scope of the present paper concerns comprehensive in-depth research into the personal experiences of informal caregivers for older adults with dementia, as well as the impact their caregiving role has on certain aspects of their own lives. A previous study has analysed informal caregivers’ experiences regarding the Lithuanian system of care for older adults with dementia [23].

Our team of researchers chose a qualitative research approach in which informal caregivers’ experiences were investigated as a phenomenon, and sought to gather data by holding focus-group discussions, which would help us to obtain a more in-depth understanding of the experiences and personal insights of the participants. The study was performed in Kaunas, the second largest city in Lithuania; Kaunas is located in the center of the country and has socioeconomic indicators that are similar to the national averages. The Kaunas Regional Biomedical Research Ethics Committee approved this study on 2018-04-23 (No. BE-2-47).

### Participants

The participants were informal caregivers who were the sole individuals providing special care or nursing to cohabiting relatives with dementia who were aged 65 years or over. Inclusion criteria for the focus group participants were: the care receiving patient was 65 years of age or older; the patient was diagnosed with Alzheimer’s disease or dementia; the patient was nursed at home; caregiver was a family member (a child of the patient, spouse or other relative) or a close friend; care receiver and caregiver were living together. Exclusion criteria for the focus group participants were: the care receiving patient was undergoing an active treatment, such as chemotherapy, surgical interventions or other; the patient was nursed at the institutional caring home; the patient was receiving palliative care.

To recruit such individuals, we focused on two large public clinics in Kaunas City (which cumulatively represented the local clinics for approximately two thirds of the population of Kaunas; one of the clinics was the biggest clinic in Kaunas and has five separate large departments located throughout the city), identifying suitable patients who were served by these clinics and, by extension, their informal caregivers.

As the first step of our research, we contacted family physicians and psychiatrists affiliated with the two large primary healthcare centers and informed them of our study. We then asked them to identify eligible patients who were nursed by informal caregivers, and to request the caregivers’ consent to be contacted for participation in the study. Consequently, a list of all informal caregivers (n = 98) who agreed to participate in the study was created. Our researchers then contacted the informal caregivers via telephone and invited them to participate in the study. A total of 94 informal caregivers were invited to participate (four were not invited as a result of technical difficulties, such as an incorrect telephone number listed);of these, 31 agreed. Among the most frequent reasons for refusing to participate were difficulties regarding leaving care receivers alone and an inability to find a replacement carer; other reasons included unwillingness to discuss the care receiver in a group discussion with other participants, and poor health on the part of the caregiver. Participation was on a voluntary basis; no reward for participation was offered or provided.

The participants were informed of the objectives and the course of the study, as well as their rights and entitlement to terminate their participation at any time. Participants then signed an informed consent form and completed an anonymous questionnaire that obtained details such as their age, gender, relationship with the care receiver, and the length of time they had been providing care. For confidentiality reasons, participants were asked not to mention any given or family names during the discussions.

### Data collection

Five semi-structured focus-group discussions were held in January–April 2018. Each focus group comprised 5–9 participants and two researchers. Five participants took part in the first focus group, six in the second, nine in the third, five in the fourth and five in the fifth. Groups were homogenous whereas all participants provided daily caregiving for a dependent person with dementia and all of them were family members of younger generation (e.g. daughters, sons). Each discussion was audio-recorded. The structure of the discussion was monitored by the researchers, who asked four pre-prepared open questions, but the participants could also discuss other aspects and were encouraged to share any experiences they thought were relevant. The four prepared questions were: (1) “What type of specialists/individuals do you think are directly involved in the care/supervision or coordination of the person you care for?” (2) “How would you evaluate the present care of the person you nurse—can you name any positive and negative aspects?” (3) “In your opinion, what are the basic needs of your care receiver that, if met, would ensure proper, more effective, and better care, overall?” (4) “What is your personal experience as a caregiver? “At the end of each discussion, we asked an additional question: “Are there any other aspects of your experiences that have not been discussed that you think are important to mention?” The focus-group discussions lasted 45–70 min, depending on the size of the group and the depth of discussion that occurred in response to the additional information provided by participants. Saturation of the theme was reached after the fourth focus-group discussion, as the fifth focus-group discussion provided no new content; consequently, the data collection was stopped after the latter discussion.

### Data analysis

After the five focus-group discussions had been completed, the audio recordings were transcribed verbatim. Two independent researchers systematically reviewed the entire dataset and coded the data by selecting the closest coding terms for the words used by the participants. Later, each researcher’s coded transcripts were compared with the others, and this showed that the majority of the codes were similar. Any coding differences that were found were resolved through securing common consent among the researchers. Thematic categories were composed by grouping similar codes, and these were then formulated into main themes. The data analysis was based on the inductive approach and the thematic analysis strategy [24] with the aim of effectively identifying and summarizing the problems experienced by the participants.

In the quotations presented in "Results" section we have used several symbols: bracketed ellipses ([…]) indicate parts of a sentence that have been omitted; for ordinary or significant pauses we have inserted ellipses (…); and for the investigators’ explanations we have used square brackets (e.g., “[address]”). Additionally, labels have been inserted to indicate the source of the citations in relation to the original datasets (e.g.,“G3” indicates the third focus group).

## Results

There were 31 participants in this study (Table 1). The majority were female (n = 28),with most being daughters of the care receivers (n = 20). The youngest caregiver was 34 years old, and the oldest was 74 years old (interquartile range: 48–58 years). The longest time spent caring was 26 years, and the shortest time was one year (interquartile range: 2.5–8 years).

**Table 1:** Demographic data of the study participants

Gender (n)	
Women	28
Men	3
Age (years)	Min 34; max 74 IQR*: 48–58
Relation to the care receiver (n)	
Daughter	20
Niece	4
Granddaughter	2
Son	2
Daughter-in-law	1
Son-in-law	1
Not a relative	1
Years of caregiving	Min 1; max 26 IQR*: 2.5–8
Age of the care receiver (years)	Min 67; max 98 Average: 85.6
Gender of the care receiver (n)	
Females	31
Males	4
Primary mental disorder (n)	
Dementia	31
Alzheimer’s disease	4

*IQR: interquartile range

Through the data analysis, the researchers identified four thematic categories relating to the personal experiences of the participants: (1) learning caregiving through personal experience; (2) implications of caregiving on social wellbeing; (3) caregivers’ contradictory emotions regarding care delivery; and (4) addressing challenges regarding care provision.

### Learning caregiving through personal experience

The informal caregivers developed a set of specific care competencies over the course of their caring practice, ranging from logic-based organization of care (e.g., segmentation of hygienic procedures—“She gets tired during the day. First, I wash her head; I wash the rest of her body the following day” G5) to nursing-care provision (e.g., experience in the management of leg ulcers) and dietary supplements (“I give her a variety of vitamins”; G2). Some focus-group participants indicated their active involvement in the adjustment of treatment (certain self-medication behaviour towards medication administration), which may change the initial treatment prescribed by the physician:

She [the care receiver] was prescribed sticking patches [for pain management]. These had some effect, but she developed spasms […], so I decided to discontinue the treatment and removed the patch. Maybe there was an overlap of medicines? […] later, they prescribed morphine. Once her pain had subsided, she no longer needed the morphine, so I stopped giving it to her. […] for Parkinson’s […] she takes two kinds of medicine—two tablets in the morning and two in the evening. On days when I notice that she is not trembling, I reduce her intake by one tablet. Then, I give her pills for dementia, for her confusion, which I cut in half [regulates the dose] (G4).

The focus-group participants reported experiencing anxiety, as a result of their relative inexperience, when interacting with existing care resources, which led to inadequate usage of these resources—“one does not know to what one is entitled—what we can get” (G1).

We […] learned too late that there are such things as [day] centers. That a bus comes and takes them [the care receivers]. They can draw and write there; they need communication (G2).

The caregivers also expressed worries regarding deficiencies in their caregiving skills and felt that there is a need for formalized educational activities to help them address various issues that arise during caregiving.

Well, I am doing something here [providing care]. However, you do something and sometimes wonder whether you are doing it well […]. But you cannot know everything. If, somehow, you could be instructed, could get basic training […], have someone explain it plainly to you. Maybe […] there is some kind of methodology [for providing certain types of care] […]. They [formal caregivers] could give demonstrations and say “do this” or “do that” […] (G2).

Caregiving competencies were obtained through many means. The focus-group participants indicated that experience, even if fragmented, was the most widespread means of gaining a better understanding of the care process.

You do not even know what you need, in the sense that there are many types of tools [for caregiving].

I cannot even imagine what else is needed.

Unless you have already [cared for] a person, you really do not know [what to do when providing care]. It [the caregiving] seems to be ok as it is (G4).

The discussion revealed that the participants attached a high level of significance to medical professionals’ roles in terms of providing information, advice, or tutorials concerning the pathogenetic disease process, care strategies, and the administering of specific procedures: “they showed us exercises and told us what to do” (G4). Social communication with peers also seemed to be an important source of information that increased the participants’ knowledge regarding care resources: “I only learned of it from other people” (G1).

Informal caregivers’ educational backgrounds and professional experience in the medical field seemed to have a strong effect on their care approach, which indicated that these factors could represent important considerations in regard to enhancing the organization of informal care and improving the results of nursing care.

My mother… she had a stroke three years ago. She is 82 now. So, she could not speak or walk and could not communicate. I am a speech therapist, so I began teaching her [to speak] step-by-step, one syllable after another. And now […] I can at least understand what she wants to say (G3).

You know, I am a nurse anaesthetist. So, I can do infusions, if I think one is needed, or give some medication […]; maybe it is easier for me. I consult with the doctor, he gives me prescriptions, and I administer them (G5).

### Implications of caregiving on social wellbeing

Providing care for older adults with dementia is often a permanent, 24-h job that substantially affects the daily life of the caregiver: “I have practically no personal life” (G4). The participants emphasized that caregiving involves being on virtually permanent standby, including during the night.

You have to listen all the time. Always be on standby (G4).

The nights were probably the hardest (G3).

Participation in the job market while performing caregiving is virtually impossible as a result of the absence of a supporting network for caregivers in Lithuania. Exclusion from the labour market has a negative effect on caregivers’ income and also shortens their work record, which will eventually have an impact on their income in the future, as the pension provided by the state depends on the years spent in the labour market.

I am delighted that I can be with my mom all the time, but I am also very sad that it is a job. It is my job. And, you know, it is hard work, exhausting. But I do not receive a salary and cannot include it on my work record (G3).

Sad, very sad […], because my years of work experience were all for nothing and… What will I get as a pension? It is ok, for now, there is my mother’s pension […] but later? What will I do? (G1).

### Caregivers’ contradictory emotions regarding care delivery

During discussions, the focus-group participants expressed a broad spectrum of emotions, often ambivalent, ranging from indifference—“just living, and that is it” (G5)—to highly positive or negative emotions.

I really find it hard. And I love my aunt, she is family. But, sometimes coming home… it seems… you do not want to do it [provide care] anymore. It is like that; that is my experience (G5).

The study participants frequently mentioned their love for their parents and grandparents, and that their caregiving gave them a chance to return the care they had received during their lifetimes and to strengthen existing relationships: “[…] we are pushed into some kind of circle. […]…the love for our parents does not end” (G2). Informal caregivers sometimes perceived, based on the changing dynamics of their relationship, caring for their loved ones as evidence of their maturity.

When a person you are close to, your mother, who was an example to you during your lifetime—she lived an active life, did such miraculous things … I thought, “God,, I could never do the things she did” … And then she became disabled. Then, I felt pity for myself; that it was over; I was no longer a child. Sometimes I would ask her, “Do you know who I am?” She would say—“Yes, I know … you are my daughter.” Am I your daughter or am I your mommy now? (G2).

The study participants had a common perception that they were of absolute importance in the lives of the care receivers—“I am her only contact with the outside world in every regard” (G3). Sometimes, sense of complete competence or even omnipotence in relation to care was expressed: “I cannot trust the care of my mom to anyone, because I know that nobody will look after her better than I do” (G5). On the other hand, the focus-group participants also highlighted negative feelings, such as isolation, loneliness, and anger.

My emotions … they change suddenly … I become angry. Sometimes, I feel unwillingness and apathy for no reason at all (G2).

The participants often expressed a sense of suffering as a result of being completely restricted, or even trapped, by their situation: “Like a prisoner; actually, my mother’s hostage” (G4). Some focus-group participants reflected on their own lives, which were dominated by, or even neglected to maintain, their caregiving role.

For example, I cannot work normally. I cannot go to work, I have lost my income because I cannot leave her. […] it is not normal. From a moral point of view, yes, it [providing care] is a child’s obligation…but she is 65 and I am 42 […]. But that is life; you have to make sacrifices (G5).

Such emotional tensions relating to caregiving could also lead to a deterioration of mental and physical health. Holding responsibility for the care often means that caregivers’ individual needs are neglected, and this can also be accompanied by feelings of anxiety and blame that not everything possible regarding caregiving is being done.

You become furious for a moment, but then you yell and yell […]. I unload my emotions because they [doctors] told me to. You should not keep them inside, because your blood pressure will go up to 200. My cardiologist said so—“you can yell.” (G2).

When she had to go to a nursing hospital, she [the care receiver] said that we [the caregivers] wanted to get rid of her (G2).

Caregiving is highly demanding and causes one to reallocate priorities relating to personal and family life, which can lead to conflicts and a deterioration of family relationships.

My husband feels very angry and annoyed about the situation, but […], who else should provide care, if not the relatives […] (G5).

### Addressing challenges regarding care provision

The focus-group participants shared their experiences regarding coping with the challenges of caregiving. Adopting a positive attitude towards the situation, securing temporary respite, finding time for communication with other people, and ensuring one’s privacy were reported to be essential measures for the prevention of burnout.

It is a must, a must, that someone substitutes for you for some time on at least one day a week, Saturday or Sunday, or else you will become sick (G5).

On the other hand, study participants also emphasized the importance of support from family members, and from health- and social-care professionals.

My husband and I provide care together, so it does not become too difficult. We let each other take time off when needed. But, if you are alone, then it is very hard (G5).

The nurse really helped by giving advice […]. When you get such support, how can I put it? You feel better (G2).

When discussing means of enduring the complex situations associated with caregiving, the participants emphasized the need for professional help from psychologists. Furthermore, it also seemed that support groups could exert a positive influence.

My opinion now is that if you see a person who is providing care for a person with disabilities […] who needs special needs or nursing, you must also ask about the condition of the relatives. The people who are living in the same home, and who are also caregivers. “How are you?” […] “Do you need any help?” […] yes, we do find other caregivers [to substitute for a time], we struggle through […]. But our psychological life […]; we should visit a psychologist or somebody (G2).

I think everyone needs a psychologist. And I probably do, too (G5).

After sitting here [in the focus-group discussion] and listening… Jesus, thank God that I am not the only one living like this (G1).

We talked, we complained [during the focus-group discussion]. No one else would have understood (G5).

Caregivers emphasized the difficulties they experienced when communicating with professionals from the health-care system. Informal caregiving has not been integrated into the Lithuanian healthcare system and is somewhat disregarded—the focus-group participants did not feel like members of the health- and social-care network, but rather as isolated individuals fighting against the network. They mentioned several hurtful interactions with the health-care system.

If such a situation occurs [the need for hospital treatment], we go to the emergency department. And there … it is scary. You are left alone; you are completely alone and the situation there is terrible for you […] because the attitude is outrageous. […] And if you show a little bit of knowledge and stand up for the person [the care receiver], you immediately receive negative comments and questions: “maybe you are a doctor, since you are asking all these questions?” Specifically, in my case… I usually say, “I am not a medic; however, I nurse my two parents, so I need to be somewhat interested in medicine.” […] If you find a humane doctor, only then will you find some compromise (G2).

I am feeling tired because I have not had a vacation […] for nine years; not a single day off. Really. … She [the care receiver] was admitted to the hospital and the doctor [said]: “you just want to have a vacation.” No support, just negative comments… (G5).

## Discussion

The present study, examining informal caregivers of older adults with dementia, revealed a variety of experiences, contradicting emotions, and problem-solving strategies relating to caring for such individuals. Four main themes of informal caregivers’ experiences were identified: (1) learning caregiving through personal experience; (2) implications of caregiving on social wellbeing; (3) caregivers’ contradictory emotions regarding care delivery; and (4) addressing challenges regarding care provision.

Our findings show that informal caregivers obtain their caregiving knowledge through personal experience and social networks. They seldom receive instruction from health-care providers, and are highly involved in care management, including the correction of medication regimes. However, cases when they must exceed the limits of their competence may result in negative implications regarding the quality and outcomes of care. Moreover, fear of incompetence and ignorance regarding care resources can also evoke psychological distress among caregivers. The focus-group participants expressed a need for formalized training relating to caregiving; several previous studies have reported similar findings: a study performed in the United States revealed that a caregiver-support program provided caregivers with valuable clinical knowledge and essential skills [25]; while other studies have emphasized the benefits of training formal caregivers by teaching them basic care skills, introducing potential care-related resources, and providing information regarding support services [26].

Our results showed that the caregivers’ social wellbeing was negatively impacted by their difficult work situations. The development of caregiver-training programs should also facilitate caregivers’ inclusion as members of the health- and social-care team, as their input in such care is essential. Further, specific policies that acknowledge caregivers’ work, facilitate their work-life balance, and prevent further social inequality are needed [20, 27]. Our study participants expressed anxiety regarding their future; in Lithuania, the time one spends excluded from the labour market performing informal caregiving is not included in his/her work record (in contrast to the time spent on maternity leave),which might affect future retirement plans (as less time in the work force means lower state-provided pension payments). Previous studies have indicated that workplace and social policies, such as access to paid family leave, can buffer the negative financial impacts of caregiving [15].

The present results suggest that becoming a caregiver can cause a significant transformation in one’s personal life, including a change in work-life balance and family relationships and a deterioration of emotional and physical health. These findings are consistent with the results of previous studies [28]. Although the focus-group participants reported contradictory and ambiguous feelings regarding their situations, many positive aspects related to care were revealed: love, possibility of returning the care one had received over his/her lifetime, perception of the importance of one‘s own role as a caregiver, personal growth and maturity, and a sense of competence. Such positive reflections have a beneficial effect on the well-being of informal caregivers, and interventions that enable caregivers to gain further positive experiences in their roles should be encouraged [9, 29]. Moreover, taking into account that informal caregiving is an essential component of any welfare ecosystem in the world, it would be the time to consistently involve these aspects into an actual discourse that could contribute to a more positive social positioning of informal caregiving. The focus-group discussions served as a means for reflection, expressing emotion, and information-sharing. It is highly probable that peer-support self-help groups can provide similar opportunities for caregivers. Engagement in social activities and self-help groups have previously been found to buffer the negative impact of caregiving [30, 31]. Possible approaches for reducing high levels of psychological distress and caregiver burden could include providing peer and professional psychological support [32], enabling caregivers to gain more positive experiences while caregiving and enhancing their sense of coherence [11, 29], and helping caregivers to stay engaged in the workforce [15]. In particular, improving caregivers’ access to peer support, in conjunction with providing internet-based intervention programs, could form an essential part of a comprehensive approach targeting informal care resources [32]. Participants of the aforementioned United-States-based caregiver-support program described the caregiver support groups as a lifeline and a source of much-needed psychological support [25]. Additionally, it should be emphasized that studies have suggested that non-financial measures, such as leisure time, psychological assistance, and training in nursing, might have a greater preventive effect on caregivers’ health than material support [6, 33].

The growing concern regarding the informal approach towards delivering long-term care means that a reshaping of existing care systems is necessary. First, the need for formalization of informal caregivers’ status has become evident [20]. Several studies conducted by the European Commission have examined various legislative aspects of issues relating to informal caregivers, such as work-life balance and the quality of care provided [4, 20]. Second, there is a need for more consistent involvement of informal caregivers in continuous care; their need for training through structured programs has been widely discussed in existing research [3, 18, 33, 34]. Lastly, potential consequences, such as social and gender inequalities, of the expansion of informal caregiving should be adequately addressed, and protective policies for minimizing the risk of caregiver overburden should be implemented [15, 27].

The present research revealed personal and sensitive details regarding the experiences of informal caregivers of older adults with dementia. A more in-depth analysis of this area would be useful for fostering the improvement, development, and organization of care for older adults, which should accommodate the needs of both the care receivers and the informal caregivers.

Research on long term care for older patients with dementia is still missing not only in Lithuania but in other Eastern European countries as well [35]. Reflection on cultural and historical backgrounds would also be indisputably valuable as differing courses of development regarding health- and social-care-service structures can shape different expectations and needs concerning the care of older adults, as well as regarding the interventions required. Research shows that dementia prevalence rate in Eastern and Western Europe are similar [36], and our study suggests that the caregiving experiences are similar as well [37, 38]. This might open the door to higher applicability of efficient tools for improving dementia care from Western societies to the Eastern European context.

### Limitations

This study has several limitations. Our participants were approached through family physicians and psychiatrists; therefore, it is possible that informal caregivers of patients who are on the records of the social welfare sector would have reported different experiences.

Data suggests, that typical care providers for patients with dementia are their spouses or children [39]. However, almost all of our study participants were from younger generation of care receivers. This random but specific sample might be due to the care receivers older age (the mean age of the care receivers in our study was 85.6 years of age, meanwhile life expectancy of the Lithuanian population is 74.6 years [40]). Spouses of such patients are usually in need of the care themselves because of their own state of health (i.e. two focus group participants cared for both parents with dementia). Spouses providing caregiving for patients with dementia might have had a different experience, therefore our data might not reflect the full range experiences of a typical dementia caregiving.

Further, we may also have missed some of the more negative experiences of target informal caregivers, as some of the caregivers we approached were unable to participate in the study as a result of an inability to find a replacement caregiver for their care receiver.

## Conclusions

Becoming a caregiver involves professional and psychological development, but also requires a relevant educational background, a positive attitude, and a supportive environment. Furthermore, this role can foster contradictory feelings and differing problem-solving strategies regarding the care of older adults with dementia. Informal caregiving largely impacts caregivers’ emotional, physical, and social wellbeing. Therefore, in order to ease the burden of caregiving and prevent burnout and social disadvantages, a comprehensive approach to supporting such individuals is needed.

## Data Availability

The datasets generated and/or analyzed during this study are available from the corresponding author on reasonable request.

## Abbreviations

G1: First focus group
G2: Second focus group
G3: Third focus group
G4: Fourth focus group
G5: Fifth focus group
